# The moderating role of father involvement in the association between maternal depression and child nutrition: A cross-sectional study in rural Malawi

**DOI:** 10.1371/journal.pone.0336485

**Published:** 2025-12-04

**Authors:** Suhyoon Choi, Yaeeun Han

**Affiliations:** 1 Centre for Family and Population Research, National University of Singapore, Singapore, Singapore; 2 Department of International Studies, Kyung Hee University, Seoul, South Korea; Christian Medical College, INDIA

## Abstract

This study highlights the critical role of fathers in improving child dietary diversity in low-resource setting, particularly in the context of maternal mental health challenges. Using cross-sectional data of 333 mother-father-child triads in rural Malawi, we examine the association between paternal involvement and child dietary diversity among children aged between 24–60 months. Our findings show that greater father involvement is significantly associated with higher child dietary diversity scores, even after accounting for maternal depressive symptoms. Importantly, we identify a significant interaction between father involvement and maternal depressive symptoms: paternal engagement buffer the negative effects of maternal mental health challenges on children’s diets. However, father involvement does not correlate with household-level dietary diversity, suggesting that paternal contributions are targeted specifically toward children- primarily through the reallocation of nutrient-rich, animal-sourced foods. These results underscore the importance of culturally sensitive, family-centered nutrition interventions that actively engage fathers while addressing maternal mental health to improve child nutrition outcomes in low-resource contexts.

## Background and context

Low dietary diversity remains a persistent challenge in addressing child malnutrition, particularly in low- and middle-income countries (LMICs) [1–2]. In Malawi, 37% of children under five years are stunted – a widely recognized marker of chronic undernutrition – with 3% underweight and about 12% wasted [3]. Children’s dietary diversity, an important indicator of diet quality, is strongly associated with improved health, growth, and cognitive development [4–6]. However, factors such as food insecurity, socioeconomic disparities, and entrenched cultural norms contribute to inadequate dietary diversity among children [7–10]. In many LMICs including Malawi, children’s diets remain predominantly reliant on starchy staples, with limited access to nutrient-dense foods like animal-sourced products and oils [11,12]. These challenges are compounded by intra-household food allocation dynamics [13–15] and the mental health status of primary caregivers, particularly mothers [16–18].

To address these challenges, it is crucial to examine how family dynamics, including the roles of both fathers and mothers, influence dietary diversity [19]. While maternal caregiving has traditionally been the focus of child nutrition research, emerging evidence underscores the significant, yet often underexplored, role fathers play in shaping children’s diets [20–22]. Fathers impact dietary diversity not only through their control over household finances and food purchases but also through mealtime behaviors and decisions about food allocation [23,24]. Studies show that fathers often receive the largest portions of nutrient-dense foods, such as animal-sourced products, reflecting cultural norms that prioritize fathers as household heads [25–27]. However, when fathers take an active interest in childcare, this dynamic can shift. Engaged fathers have been shown to reallocate high-value foods toward children, improving their dietary outcomes [24,28,29]. For example, studies found that paternal involvement in food-related decisions is associated with increased consumption of animal-sourced foods among children in sub-Saharan African countries [24,29].

The potential of fathers to influence child nutrition becomes even more significant when maternal mental health is considered. Maternal depression, a pervasive mental health issue in LMICs, affects 19–25% of women during the perinatal period [30]. Depressive symptoms in mothers have been strongly linked to reduced dietary diversity, undernutrition, and stunting in children. For example, Surkan et al. (2011) found that maternal depressive symptoms significantly increased the likelihood of child underweight and stunting [31], while Miller et al. (2021) reported that maternal depression in Nepal was associated with an 11% lower likelihood of children consuming diverse diets, particularly nutrient-dense foods such as animal-sourced products [32]. These negative effects are likely due to the reduced caregiving capacity of depressed mothers, who may struggle with responsive feeding practices or lack the energy and motivation required for meal preparation.

In such contexts, fathers have the potential to buffer the negative effects of maternal depression on child nutrition. By stepping into caregiving roles and participating in food-related decisions, fathers may mitigate the gaps caused by maternal mental health challenges, ensuring that children’s nutritional needs are met.

Although recent interventions in sub-Saharan Africa and other LMICs have begun to incorporate fathers into child nutrition and caregiving programs—with some reporting improvements in dietary diversity, responsive feeding, and caregiver well-being—the evidence remains mixed and limited in important ways [33–35]. For example, a recent cluster-randomized trial in rural Ethiopia found that engaging fathers through nutrition behavior change communication **did not improve** child dietary diversity, highlighting the complexity and context-dependence of father-focused interventions [3]. Furthermore, the evidence base remains limited in important respects. Many prior interventions have been *multicomponent* or “bundled” – combining father engagement with other interventions – which makes it difficult to isolate the specific effect of paternal involvement on child nutrition [33]. Moreover, very few studies have examined how father engagement might interact with maternal mental health to influence child feeding outcomes. As a result, despite growing policy interest in engaging fathers, our understanding of the exact pathways through which fathers affect child dietary outcomes – particularly in contexts of high maternal psychological distress – remains underdeveloped

This study seeks to fill that gap by examining whether paternal involvement in childcare is associated with child dietary diversity and whether it moderates the relationship between maternal depressive symptoms and child nutrition in rural Malawi. By using cross-sectional data from a sample of mother–father–child triads, this research distinguishes between child-specific and household-wide dietary impacts and explores intra-household food allocation dynamics. In doing so, it contributes new empirical evidence to a limited but growing body of literature on the role of fathers in child nutrition, particularly under conditions of maternal vulnerability. The study’s findings have implications for designing more targeted, family-centered interventions in low-resource settings where both food insecurity and maternal mental health burdens are high.

## Methods

### Study setting and participants

This cross-sectional study was conducted between June 24th and July 25th 2019 as a baseline survey for a parenting education program targeting caregivers in the Traditional Authority Chimutu, a rural district in Malawi. The study district shows some of the poorest growth and developmental outcomes for children under five, with 35% of children stunted and 45% showing signs of suspected developmental delay, based on our baseline data collected using the Early Childhood Development Index. The parenting program aimed to enhance parenting practices to promote early childhood health and development.

We employed a two-stage stratified sampling design to select participants from the Traditional Authority of Chimutu. The program initially enrolled 427 mothers with children aged 24–60 months from 20 group villages. For this study, we restricted our analytical sample to mothers who were formally married or co-residing fathers in the same household for at least for the past 12 months (N = 333).

As this study used data from a baseline survey of a community-based intervention, no formal sample size estimation was conducted prior to data collection. However, to assess the statistical sensitivity of the sample, we conducted a post hoc power analysis for the primary outcome. With 135 participants in the “father involved” group and 198 in the “father not involved” group, the study had approximately 89% power (α = 0.05) to detect the observed mean difference of 0.45 in child dietary diversity scores (SD = 1.27).

### Data collection

Trained enumerators collected baseline data. The survey included questions to assess father involvement in childcare, maternal depressive symptoms, dietary patterns of children and households, and sociodemographic indicators (See S1 Table). The survey was translated into Chichewa, the local language, by the research team, then back-translated into English to ensure accuracy. Pre-testing of the surveys was conducted to confirm the clarity and cultural appropriateness of the questions for the rural Malawian context. Data were collected using CommCare, an app-based data collection tool.

### Ethical considerations

The study was approved by the National Health Sciences Research Committee (NHSRC) on January 28, 2019 (Approval Number: 2150) and was conducted in accordance with relevant ethical guidelines. Written informed consent was obtained from all participants. Data collection was led by the first author in Malawi through the Africa Future Foundation, with study design developed by the authors and funding from KOICA. Ethical approval and fieldwork were conducted in collaboration with local partners.

### Measurements

#### Dependent variables.

*Child dietary diversity score* (CDDS) was assessed using a locally adapted food frequency questionnaire containing 25 food items the child aged 24–60 months consumed in the past 24 hours [36,37]. The food items were aggregated into eight groups: 1) starchy staples, 2) vitamin-A-rich fruits and vegetables, 3) other fruits and vegetables, 4) flesh foods, 5) eggs, 6) legumes and nuts, 7) dairy products, and 8) fat and oils. The scores for each group were then summed up to obtain the CDDS, ranging from 0 to 8, with higher scores indicating greater dietary diversity.

The *Household Dietary Diversity Score* (HDDS) is a proxy indicator of household access to a variety of foods [38]. The HDDS was measured using a household food frequency questionnaire, including commonly consumed food items in Malawi. Mothers reported the frequency of food items consumed by the household over the past week. The food items were categorized into 12 groups based on the Food and Agriculture Organization guidelines: 1) cereals, 2) roots and tubers, 3) vegetables, 4) fruits, 5) meat, poultry, and offal, 6) eggs, 7) fish, 8) pulses, legumes, and nuts, 9) milk and milk products, 10) fat and oils, 11) sugar, and 12) condiments. A score of one was assigned if a household consumed a food group at least once during the week. The HDDS was calculated as the total number of food groups consumed by the household, with a possible range of 0–12. Higher scores indicate greater dietary diversity.

The *Household Food Consumption Score* (FCS) was calculated using a 7-day food frequency questionnaire combined with a weighted scoring system that reflects the nutrient density of different food groups [39]. The FCS serves as a proxy measure of household caloric availability. Food items were categorized into eight groups: 1) main staples, 2) pulses, 3) vegetables, 4) fruits, 5) meat and fish, 6) dairy products, 7) sugar, and 8) fat and oils. The FCS was calculated by summing the weighted scores assigned to each food group.

#### Independent variables.

*Father’s involvement in childcare* was assessed using the Father’s Child Care Involvement Scale, reported by mothers and partially modified to fit the cultural context [40]. The scale consists of four items: 1) “Father buys toys and other items for our child,” 2) “Father takes an interest in our child’s habits and behaviors (didactic),” 3) “Father feeds or bathes our child (caregiving),” and 4) “Father plays with our child (physical play).” The number of ‘yes’ responses was summed across items, resulting in a score ranging from 0 to 4, with higher scores indicating greater involvement. The scores were then categorized into a dummy variable: *father involved* (score 1–4) and *father not involved* (score 0).

*Maternal depressive symptoms* were assessed using the Patient Health Questionnaire Depression Scale (PHQ-8) [41]. Mothers responded to eight items regarding how often they had experienced the following symptoms over the past two weeks: (1) little interest or pleasure in doing things, (2) feeling down, depressed, or hopeless, (3) trouble falling or staying asleep, (4) feeling tired or having little energy, (5) poor appetite or overeating, (6) feeling bad about oneself, (7) trouble concentrating, and (8) moving or speaking slowly. Responses were rated on a 4-point Likert scale, from “0 = not at all” to “3 = nearly every day,” with total scores ranging from 0 to 24. The PHQ-8 demonstrated acceptable internal consistency, with a Cronbach’s alpha of 0.79.

#### Control variables.

We include baseline characteristics of mothers, children, fathers, and households that have been shown to be associated with child dietary diversity in previous literature [24]. The models control for the mother’s age, mother’s educational attainment (completed high school vs. not), mother’s participation in non-agricultural labor, child’s age (in months), child’s sex, whether the child has siblings, and father’s employment status. Additionally, we control for whether the household owns books for children and has access to improved sanitation facilities. We used the variance inflation factor (VIF) to check for multicollinearity among the independent variables and the mean VIF was less than 5, indicating the absence of significant correlation [42].

### Data analysis

We conducted all statistical analyses using STATA version 15.1, setting statistical significance at a p-value of less than 0.05. Descriptive statistics were used to summarize the characteristics of mothers, fathers, children, and households. Dietary patterns were described using means and standard deviations for continuous scores and proportions for binary food group items. Bivariate analyses compared dietary diversity patterns by father involvement using chi-square tests for binary variables (food group items), Welch’s t-tests for continuous outcomes (CDDS and FCS), and the Wilcoxon rank-sum test for skewed variable (HDDS).

We performed two-level mixed models with random intercepts to account for the clustering of households at the village level. Ordinary least squares regression was used for continuous outcome variables: CDDS, HDDS, and FCS. For each outcome, we first fitted models with father involvement and control variables. In the second set of models, maternal depressive symptoms were added to the initial model. Finally, we included an interaction term between father involvement and maternal depressive symptoms to examine whether the relationship between maternal depressive symptoms and nutrition outcomes varied based on father involvement. Additionally, we assessed the associations between father involvement and children’s diets by food groups using binomial logistic regressions.

## Results

The mean age of mothers was 29.7 years (median: 29; IQR: 24–35), with 70.3% being literate (Table 1). Regarding occupation, 39.9% of mothers were engaged in farming, while 29.2% identified as homemakers. 86.3% of mothers reported as Christian. Fathers had a mean age of 34.5 years (median: 34; IQR: 28–40), and 87.4% were literate; among them, 44.4% worked as laborers, and 33.6% were farmers. The mean age of eligible children was 43.2 months at baseline (median: 43; IQR: 39–48), and 75.2% had at least one sibling. In 96% of households, mothers were identified as the primary caregivers, and 38.2% of households were classified as having poor food consumption status measured by food consumption score.

**Table 1:** Household characteristics.

	Mean (SD) or %
**Mother’s characteristics**	
Mother’s age (years)	29.7 (6.7)
Mother’s literacy (yes)	70.3
Mother completed high school or higher	16.5
Mother’s primary occupation	
Homemaker	29.2
Farmer	39.9
Non-agricultural laborer	30.9
Mother’s religion	
Christian	86.3
Roman Catholic	10.2
Other	2.4
None	1.1
**Father’s characteristics**	
Father’s age (years)	34.5 (8.1)
Father’s literacy (yes)	87.4
Father completed high school or higher	36.1
Father’s primary occupation	
Unemployed	3.3
Farmer	33.6
Laborer	44.4
Self-employed	18.6
Paternal involvement sub-score	
Caregiving (feeding and bathing)	7.6
Physical play	13.7
Didactic	33.2
Buying toys and things	3.2
Father involved (yes if total score = 4)	39.4
**Child’s characteristics**	
Child’s age at baseline (months)	43.2 (5.6)
Child’s sex (girl)	50.2
**Household characteristics**	
Female household head (yes)	4.7
Whether the child has siblings (1 = yes)	75.2
Primary caregiver	
Mother	96.2
Father	2.3
Grandmother	0.3
Older sibling	0.9
Other relatives	0.3
Household food consumption status	
Poor	38.2
Borderline	60.6
Acceptable	1.2

To explore the differences in dietary and household characteristics based on father involvement, Table 2 presents descriptive statistics between households with high levels of father involvement and those with limited father involvement. Across all households, the mean CDDS was 4.45 (median: 4; IQR: 4–5), the mean HDDS was 8.08 (median: 8; IQR: 7–10), and the mean FCS was 22.37 (median: 23; IQR: 17.5–27). Households with greater father involvement demonstrated a higher total CDDS, with a mean difference of 0.41 food groups (p < 0.004). This difference was primarily attributed to increased consumption of oils, unhealthy snacks, and animal-sourced foods in households with father involvement. A similar pattern was observed at the household level, where higher HDDS were noted, driven by greater consumption of oils, sugar, and condiments. Additionally, the FCS indicated a higher proportion of households with limited father involvement falling into the poor FCS category, compared to households with father involvement.

**Table 2:** Child and household dietary patterns by father involvement in parenting.

	Total (N = 333)		Father involved (n = 135)		Father not involved (n = 198)		*p*-value
	Mean	SD	Mean	SD	Mean	SD	
**Child dietary diversity**							
Child dietary diversity score (0–8)	4.45	1.28	4.70	1.22	4.29	1.30	**0.004**
Starch	1.00	0.00	1.00	0.00	1.00	0.00	–
Vitamin A-rich fruits and vegetables	0.86	0.35	0.86	0.35	0.86	0.35	0.910
Other fruits and vegetables	0.85	0.35	0.89	0.32	0.83	0.39	0.114
Meat and fish	0.31	0.46	0.36	0.48	0.28	0.45	0.166
Eggs	0.08	0.28	0.09	0.29	0.08	0.27	0.797
Legumes	0.72	0.45	0.74	0.44	0.70	0.46	0.439
Milk	0.11	0.31	0.1	0.31	0.11	0.31	0.831
Oils	0.52	0.50	0.66	0.48	0.42	0.49	**0.000**
Animal-source foods (meat, fish, egg)	0.41	0.49	0.48	0.50	0.37	0.48	**0.042**
**Household dietary diversity**							
Household dietary diversity score (0–12)	8.08	2.18	8.35	1.98	7.83	2.28	0.067
Cereal	0.98	0.12	0.99	0.09	0.98	0.14	0.305
Roots	0.74	0.44	0.78	0.42	0.72	0.45	0.175
Vegetables	1.00	0.00	1.00	0.00	1.00	0.00	–
Fruits	0.62	0.49	0.66	0.48	0.59	0.49	0.206
Meats	0.74	0.44	0.77	0.42	0.71	0.03	0.232
Eggs	0.56	0.50	0.59	0.49	0.54	0.50	0.346
Fish	0.64	0.48	0.69	0.46	0.61	0.49	0.119
Nut	0.94	0.24	0.94	0.24	0.93	0.25	0.813
Dairy	0.14	0.35	0.16	0.36	0.14	0.34	0.629
Oil	0.87	0.33	0.92	0.27	0.84	0.36	**0.033**
Sugar	0.70	0.46	0.81	0.40	0.63	0.48	**0.000**
Condiment	0.14	0.35	0.04	0.21	0.20	0.40	**0.000**
**Household food consumption**							
Household food consumption score	22.37	7.46	23.36	6.88	21.69	7.78	**0.040**

Mean and standard deviation (SD) of dietary scores and food group consumption among children and households, by father involvement. Binary food group items are coded 0/1 to indicate consumption. Group differences were assessed using Welch’s *t*-test for continuous outcomes (child dietary diversity score and household food consumption score), Wilcoxon rank-sum test for skewed outcomes (household dietary diversity score), and chi-squared tests for binary variables.

Next, to examine the relationship between father involvement, maternal depressive symptoms, and child dietary diversity, Table 3 uses ordinary linear squares regression models. The multivariate model 3a assesses the relationship between father involvement and child dietary diversity while controlling for maternal, child, and household-level factors. The multivariate model 3b examines the relationship by including both father involvement and maternal depressive symptoms as predictors. The model 3c includes an interaction term to explore how father involvement modifies the impact of maternal depressive symptoms on child dietary diversity. The multivariate analysis indicates father involvement is significantly associated with improved CDDS (Model 3a: B = 0.354, 95% CI: 0.076 to 0.632). When maternal depression is included, father involvement continues to show a significant positive association (Model 3b: B = 0.343, 95% CI: 0.065 to 0.621). Furthermore, the interaction term between father involvement and maternal depressive symptoms is significant (Model 3c: B = 0.098, 95% CI: 0.009 to 0.187), suggesting that father involvement may mitigate the negative impact of maternal depressive symptoms on CDDS. Table 3 highlights the buffering role fathers can play in supporting child nutrition when mothers experience depressive symptoms. Notably, mothers who have completed high school are positively associated with improved CDDS. These findings underscore the importance of paternal engagement in improving children’s diet and suggest that fathers may play a crucial role in addressing the challenges posed by maternal depressive symptoms.

**Table 3:** Associations between father involvement, maternal depressive symptoms, and child dietary diversity score.

	Model 3a			Model 3b			Model 3c		
	B	95% CI	*p*-value	B	95% CI	*p*-value	B	95% CI	*p*-value
Father involved	0.354	(0.076, 0.632)	**0.013**	0.343	(0.065, 0.621)	**0.015**	0.006	(-0.406, 0.418)	0.977
Maternal depressive symptom score				-0.028	(-0.071, 0.016)	0.210	-0.063	(-0.117, -0.009)	**0.022**
Father involved * Maternal depressive symptom score							0.098	(0.009, 0.187)	**0.031**
*Control variables*									
Mother’s age	0.002	(-0.022, 0.027)	0.851	0.003	(-0.021, 0.027)	0.802	0.003	(-0.022, 0.027)	0.838
Mother completed high school	0.456	(0.097, 0.815)	**0.013**	0.445	(0.086, 0.803)	**0.015**	0.472	(0.115, 0.828)	**0.010**
Child’s age (months)	0.009	(-0.015, 0.034)	0.453	0.009	(-0.015, 0.034)	0.453	0.011	(-0.013, 0.035)	0.381
Child’s sex (girl)	0.059	(-0.203, 0.321)	0.658	0.059	(-0.202, 0.320)	0.657	0.055	(-0.205, 0.314)	0.679
Child has sibling(s)	-0.100	(-0.483, 0.282)	0.608	-0.116	(-0.498, 0.266)	0.552	-0.089	(-0.469, 0.292)	0.648
Mother’s participation in non-agricultural labor	0.381	(0.093, 0.670)	**0.009**	0.391	(0.103, 0.679)	**0.008**	0.398	(0.112, 0.684)	**0.006**
Father’s employment status	0.146	(-0.139, 0.431)	0.316	0.123	(-0.163, 0.409)	0.400	0.109	(-0.176, 0.393)	0.454
Owns books	0.111	(-0.185, 0.406)	0.462	0.111	(-0.184, 0.405)	0.461	0.109	(-0.184, 0.402)	0.466
Access to improved sanitation facilities	0.193	(-0.230, 0.616)	0.372	0.203	(-0.220, 0.625)	0.347	0.207	(-0.213, 0.627)	0.334
Constant	3.404	(2.075, 4.734)	0.000	3.504	(2.168, 4.839)	0.000	3.572	(2.244, 4.900)	0.000
Group-level variance	0.088	(0.024, 0.329)		0.096	(0.027, 0.341)		0.086	(0.023, 0.325)	
Residual variance	1.422	(1.216, 1.664)		1.411	(1.206, 1.651)		1.396	(1.193, 1.634)	
Intraclass Correlation Coefficient (ICC)	0.058	(0.016, 0.192)		0.064	(0.018, 0.199)		0.058	(0.016, 0.194)	

To identify the food groups driving the relationship between father involvement and CDDS, Table 4 examines the associations between father involvement and children’s consumption by food groups, offering insights into the nature of improved diet quality; the food group that is driving the increase in CDDS. The results indicate that the significant positive relationship between father involvement and CDDS (Table 3) is primarily driven by increased consumption of animal-source foods (OR = 1.670, 95% CI: 0.021 to 1.005) and oils (OR = 2.699, 95% CI: 0.486 to 1.500), suggesting engaged fathers may specifically increase consumption of high-value, nutrient dense food groups, which is important for children’s growth and development.

**Table 4:** Associations between father involvement and child’s diets by food groups.

	AOR	95% CI	*p*-value
Animal-source foods	1.670	(0.021, 1.005)	**0.041**
Vitamin A-rich fruits & vegetables	0.841	(−0.857, 0.511)	0.620
Other fruits & vegetables	1.456	(−0.319, 1.070)	0.289
Meat & fish	1.417	(−0.149, 0.847)	0.170
Eggs	1.158	(−0.705, 0.998)	0.736
Legumes	1.146	(−0.386, 0.659)	0.609
Milk	0.838	(−0.942, 0.589)	0.652
Oils	2.699	(0.486, 1.500)	**0.000**

AOR: Adjusted Odds Ratio. Controls include mother’s age and education, child’s age and sex, presence of sibling(s), mother’s participation in non-agricultural labor, father’s wage employment, household book ownership, and access to improved sanitation facilities. *Note:* Starch was excluded from the regression analyses because all mothers in the analytical sample reported that their child consumed starch in the past 24 hours, resulting in no variation between children of involved and non-involved fathers.

To assess broader impact of father involvement on household dietary outcomes, Table 5 examines the relationship between father involvement, maternal depressive symptoms, and household dietary diversity scores. The multivariate analysis indicates that father involvement is not significantly associated with HDDS (Model 5a: B = 0.337, 95% CI: −0.140 to 0.814; Model 5b: B = 0.327, 95% CI: −0.151 to 0.804). Maternal depressive symptoms also show no significant relationship with HDDS (Model 5b: B = −0.025, 95% CI: −0.100 to 0.049). These results align with findings on household FCS, where neither father involvement (Model S2b: B = 1.545, 95% CI: −0.088 to 3.178) nor maternal depressive symptoms (Model S2b: B = 0.131, 95% CI: −0.124 to 0.386) show significant associations (S2 Table). Moreover, the interaction terms between father involvement and maternal depressive symptoms are not significant for either HDDS or FCS. This lack of association with HDDS reflects persistent traditional gender norms, where mothers primarily manage household food purchases and decisions.

**Table 5:** Associations between father involvement, maternal depressive symptoms, and household dietary diversity score.

	Model 5a			Model 5b			Model 5c		
	B	95% CI	*p*-value	B	95% CI	*p*-value	B	95% CI	*p*-value
Father involved	0.337	(−0.140, 0.814)	0.166	0.327	(−0.151, 0.804)	0.180	0.055	(−0.657, 0.767)	0.880
Maternal depressive symptom score				−0.025	(−0.100, 0.049)	0.510	−0.054	(−0.147, 0.040)	0.259
Father involved * Maternal depressive symptom score							0.080	(−0.075, 0.234)	0.312
*Control variables*									
Mother’s age	0.003	(−0.038, 0.045)	0.869	0.004	(−0.038, 0.045)	0.854	0.004	(−0.038, 0.045)	0.859
Mother completed high school	0.862	(0.247, 1.478)	**0.006**	0.850	(0.235, 1.466)	**0.007**	0.876	(0.260, 1.493)	**0.005**
Child’s age (months)	0.009	(−0.032, 0.051)	0.655	0.010	(−0.032, 0.051)	0.653	0.011	(−0.031, 0.052)	0.615
Child’s sex (girl)	−0.117	(−0.567, 0.332)	0.609	−0.117	(−0.567, 0.332)	0.608	−0.121	(−0.570, 0.328)	0.597
Child has sibling(s)	−0.400	(−1.055, 0.255)	0.232	−0.411	(−1.066, 0.245)	0.220	−0.393	(−1.049, 0.263)	0.241
Mother’s participation in non-agricultural labor	0.230	(−0.264, 0.724)	0.361	0.238	(−0.257, 0.732)	0.346	0.246	(−0.248, 0.740)	0.329
Father’s employment status	0.310	(−0.179, 0.799)	0.214	0.289	(−0.203, 0.781)	0.249	0.278	(−0.214, 0.771)	0.268
Owns books	0.277	(−0.230, 0.784)	0.284	0.275	(−0.231, 0.782)	0.287	0.275	(−0.231, 0.781)	0.287
Access to improved sanitation facilities	0.116	(−0.610, 0.841)	0.755	0.122	(−0.603, 0.847)	0.742	0.128	(−0.596, 0.852)	0.729
Constant	7.228	(4.953, 9.503)	0.000	7.325	(5.035, 9.615)	0.000	7.372	(5.083, 9.661)	0.000
Group-level variance	0.199	(0.042, 0.945)		0.208	(0.045, 0.953)		0.191	(0.038, 0.956)	
Residual variance	4.200	(3.589, 4.916)		4.190	(3.580, 4.904)		4.186	(3.576, 4.900)	
Intraclass Correlation Coefficient (ICC)	0.045	(0.010, 0.189)		0.047	(0.010, 0.190)		0.044	(0.009, 0.191)	

## Discussion

This study emphasizes the critical role of father involvement in improving children’s diets, an often-overlooked factor in nutrition research. Our findings demonstrate that increased paternal involvement is positively associated with higher child dietary diversity. Furthermore, the significant interaction between father involvement and maternal depressive symptoms suggests that fathers may buffer the negative effects of maternal mental health challenges on children’s diets.

Maternal depressive symptoms have been consistently linked to lower dietary diversity and increased risks of undernutrition in children [31,32]. When fathers are actively engaged in caregiving and feeding, they may offset these risks by sharing responsibilities, ensuring food provision, and directly supporting children’s dietary needs. Fathers also provide practical and emotional support to their partners, which can reduce maternal stress and foster more positive caregiving behaviors, thereby improving feeding practices. Evidence from similar resource-constrained contexts shows that father involvement enhances child feeding practices and diet quality [24,28,29], while also reducing maternal depressive symptoms [43]. However, the potential moderating role of father involvement in the relationship between maternal depression and children’s nutrition outcomes has been less examined.

Our findings also align with prior research emphasizing the role of fathers in enhancing child’s diet quality, particularly in low-resource settings. Studies in other sub-Saharan African countries, such as Tanzania and Rwanda, have shown that paternal involvement in food-related decisions is associated with higher consumption of nutrient-dense foods, particularly animal-source foods [24,29]. The current study builds on this literature by demonstrating that engaged fathers may actively redistribute high-value foods to their children, ensuring they receive a more diverse diet even in the face of household resource constraints.

However, our results show no significant association between father involvement and household dietary diversity. One possible explanation for this finding is financial constraints and persistent poverty in Malawi, where many households face limited access to diverse food groups due to economic hardship. Even when fathers are actively engaged in caregiving and food distribution, their influence on overall household dietary diversity may be restricted by structural economic limitations, including low household income, food price fluctuations, and seasonal food shortages [44]. This suggests that while father involvement can enhance child-specific dietary outcomes, broader household dietary improvements may require economic interventions, increased food accessibility, and policy-driven support for household food security.

The discrepancy between father involvement’s effect on child dietary diversity and its lack of association with household dietary diversity can also be understood through the lens of intra-household food allocation dynamics. In many cultural settings, fathers traditionally hold decision-making power over resource distribution within the household [45]. When engaged in childcare, fathers may deliberately prioritize reallocating nutrient-dense foods—typically reserved for household head—to their children due to their direct concern for child well-being. This targeted redistribution may improve children’s diets without significantly altering the dietary diversity of the household as a whole. Additionally, food purchasing and preparation responsibilities often remain largely under maternal control, meaning fathers may not directly influence the variety of foods available at the household level, even when they play an active role in child feeding.

This study has several limitations. First, its cross-sectional design limits the ability to assess the directionality of the relationship between father involvement and child dietary outcomes, as the temporal sequence cannot be determined. Future longitudinal studies are needed to explore the directionality and long-term effects of father involvement on child nutrition. Second, the measurement of father involvement through the Father’s Child Care Involvement Scale, which primarily focuses on instrumental caregiving behaviors, does not capture the financial contributions fathers make to child-rearing. Third, reliance on maternal reports for all assessments introduces potential biases, including social desirability bias, recall bias, and respondent interpretation differences, which may affect the accuracy of the data. Finally, the possibility of omitted variable bias exists, as unmeasured factors may influence the relationship between father involvement and child dietary outcomes, limiting the explanatory power of the findings.

## Conclusion

This study contributes to the literature by examining how paternal involvement may moderate the association between maternal depressive symptoms and child dietary diversity in a low-resource setting. Our findings indicate that father engagement is positively associated with child dietary diversity and may help buffer the adverse effects of maternal depressive symptoms. However, no significant association was found between father involvement and household-level dietary diversity, suggesting that fathers’ contributions may be more targeted toward children. These results highlight the potential value of incorporating culturally sensitive, family-centered strategies into nutrition programs that engage both fathers and mothers—particularly in contexts where maternal mental health challenges are common.

## Supporting information

S1 Table Study variable description. (PDF)

S2 Table Associations between father involvement, maternal depressive symptoms, and household food consumption score. (PDF)
