# Supplementary material for: The moderating role of father involvement in the association between maternal depression and child nutrition: A cross-sectional study in rural Malawi
Source: PLoS One. 2025 Dec 4;20(12):e0336485. doi: 10.1371/journal.pone.0336485 (PMC12677494; doi:10.1371/journal.pone.0336485)
Supplement: S1 Table — (PDF) [file pone.0336485.s001.pdf]

Supporting information

S1 Table. Study variable descriptions

**Outcome variables**		
**Child Dietary Diversity 24-Hour Recall Questionnaire**		
"I'd like to ask you about what your child [NAME] ate or drank yesterday during the day or night."		
**Food Group**	**Food Item Description**	**24-hour recall response**
Starch	Any porridge (wheat, maize/corn, barley, other grains)	1=Yes, 0=No
Starch	Any gruel (rice, oats, wheat, other grains)	1=Yes, 0=No
Starch	Commercially fortified food (Cerifam, Fafa, etc.)	1=Yes, 0=No
Starch	Bread, pasta, rice, noodles, solid foods from grains	1=Yes, 0=No
Starch	Nsima	1=Yes, 0=No
Starch	Enset, potatoes, yams, cassava, other roots	1=Yes, 0=No
Vitamin A-rich fruits and vegetables	Pumpkin, carrot, sweet potatoes (yellow/orange inside)	1=Yes, 0=No
Vitamin A-rich fruits and vegetables	Dark green leafy vegetables (mustard greens, etc.)	1=Yes, 0=No
Vitamin A-rich fruits and vegetables	Ripe papayas or ripe mangos (vitamin A-rich fruits)	1=Yes, 0=No
Other fruits and vegetables	Other vegetables (lettuce, onion, tomato, etc.)	1=Yes, 0=No
Other fruits and vegetables	Other fruits (banana, apple, citrus fruits)	1=Yes, 0=No
Meat and fish	Liver, kidney, heart, organ meats	1=Yes, 0=No
Meat and fish	Meat (beef, pork, goat, lamb)	1=Yes, 0=No
Meat and fish	Chicken, ducks, or other birds	1=Yes, 0=No
Meat and fish	Fresh or dried fish or shellfish	1=Yes, 0=No
Eggs	Eggs	1=Yes, 0=No
Legumes	Legumes (peas, lentils, beans, pulses)	1=Yes, 0=No
Milk	Milk products (cheese, yogurt)	1=Yes, 0=No
Oils	Food made from oil, fat, or butter	1=Yes, 0=No
Other	Therapeutic foods	1=Yes, 0=No
Other	Kolo, chips, crisps, popcorn	1=Yes, 0=No
Other	Candies, chocolates, cookies	1=Yes, 0=No
Other	Spices or condiments	1=Yes, 0=No
Other	Iron supplements	1=Yes, 0=No
**Household Dietary Diversity – 7-Day Recall Questionnaire**		
"Now I'd like to ask you about what your household ate over the past 7 days."		
**Food Group**	**Food Item**	**Past 7-day recall response**
Cereals	Cereals (maize, sorghum, millet, barley) or nsima or porridge made from cereals	1=Yes, 0=No
Roots and tubers	Potatoes, cassava, yam, and other roots/tubers, sweet potato, plantain	1=Yes, 0=No
Vegetables	Dark green leafy vegetables (amaranth, cassava leaves, mustard green, pumpkin greens, okra greens, spinach, amaranth leaves, spinach)	1=Yes, 0=No
Vegetables	Vitamin A rich vegetables and tubers (pumpkin, carrot, butternut, or sweet potato)	1=Yes, 0=No

Vegetables	Other vegetables (onion, tomato, eggplant, cauliflower, salata, bokchoy, cabbage, green pepper, lettuce, mushroom, okra, onion, peas/greens)	1=Yes, 0=No
Fruits	Vitamin A rich fruits (papaya, mango, anything orange inside, passion fruit, peach, tree tomato)	1=Yes, 0=No
Fruits	Other fruits (banana, peach, lemon, strawberry, baobab pulp, pineapple)	1=Yes, 0=No
Pulses, legumes, and nuts	Legumes (beans, lentils, peas, nuts)	1=Yes, 0=No
Eggs	Eggs	1=Yes, 0=No
Milk and milk products	Dairy products (milk, cheese, yoghurt)	1=Yes, 0=No
Meat, poultry, and offal	Meat (goat, beef, lamb, pork)	1=Yes, 0=No
Meat, poultry, and offal	Organ meat (liver etc.)	1=Yes, 0=No
Meat, poultry, and offal	Poultry (chicken, duck, pigeon)	1=Yes, 0=No
Fish	Fish (fresh and dry)	1=Yes, 0=No
Fat and oils	Oil/fats (ghee, butter, veg oil)	1=Yes, 0=No
Sugar	Sugar	1=Yes, 0=No
Condiments	Condiments (spices, ketchup)	1=Yes, 0=No
Pulses, legumes, and nuts	Nuts and seeds (ground nut, sunflower)	1=Yes, 0=No
Other	Alcohol	1=Yes, 0=No
Other	Tobacco	1=Yes, 0=No

Independent variables

Variable Name	Item Description	Coding
Father involvement	1. Father buys toys or things for the child (material provision)<br>2. Father takes interest in the child's habits or behaviors and instructs them (didactic)<br>3. Father feeds or bathes the child (caregiving)<br>4. Father plays with the child (physical play)	Sum of items (range 0–4). Dummy coded: 1 = Involved (≥1), 0 = Not involved (0)
Depressive symptoms	1. Little interest or pleasure in doing things<br>2. Feeling down, depressed, or hopeless<br>3. Trouble falling or staying asleep or sleeping too much<br>4. Feeling tired or having little energy<br>5. Poor appetite or overeating<br>6. Trouble concentrating<br>7. Feeling bad about oneself (e.g., feeling like a failure or disappointment to family)<br>8. Thoughts of being better off dead or self-harm thoughts	Response: 0 = Not at all, 1 = Several days, 2 = More than half the days, 3 = Nearly every day<br>Sum total score (Range: 0–24); higher scores indicate greater depressive symptoms

**Sociodemographic variables**		
**Variable Name**	**Item Description**	**Coding**
Mother's age	Respondent's age in years	Continuous
Educational attainment	Whether respondent completed high school or higher	Binary: 1 = high school or higher, 0 = less than high school
Mother's literacy	Ability to read and write a letter	1 = literate (can read and write), 0 = illiterate
Mother's participation in non-agricultural labor	Whether respondent engages in non-farm work	Binary: 1 = yes, 0 = no
Mother's religion	Religious affiliation	Categorical: Christian, Roman Catholic, Other, None
Child's age (in months)	Age of child in months	Continuous
Child's sex	Sex of child	Binary: 1 = girl, 0 = boy
Child has siblings	Whether the child has any siblings living in the household	Binary: 1 = yes, 0 = no
Father's literacy	Ability to read and write a letter	1 = literate (can read and write), 0 = illiterate
Father's employment status	Father's main occupation	Categorical: unemployed, farmer, laborer, self-employed
Father completed high school or higher	Whether father completed high school or higher	Binary: 1 = high school or higher, 0 = less than high school
Household owns books for children	Whether household owns any children's books or picture books	Binary: 1 = yes, 0 = no
Access to improved sanitation facilities	Whether household has access to an improved toilet/latrine	Binary: 1 = yes, 0 = no
