# Supplementary material for: The moderating role of father involvement in the association between maternal depression and child nutrition: A cross-sectional study in rural Malawi
Source: PLoS One. 2025 Dec 4;20(12):e0336485. doi: 10.1371/journal.pone.0336485 (PMC12677494; doi:10.1371/journal.pone.0336485)
Supplement: S2 Table — (PDF) [file pone.0336485.s002.pdf]

Supporting information

S2 Table. Associations between father involvement, maternal depressive symptoms, and household food consumption score

	Model S2a			Model S2b			Model S2c		
	B	95% CI	*p*-value	B	95% CI	*p*-value	B	95% CI	*p*-value
Father involved	1.492	(-0.140, 3.124)	0.073	1.545	(-0.088, 3.178)	0.064	0.637	(-1.799, 3.072)	0.608
Maternal depressive symptom score				0.131	(-0.124, 0.386)	0.313	0.035	(-0.284, 0.354)	0.830
Father involved * Maternal depressive symptom score							0.266	(-0.262, 0.793)	0.324
Control variables									
Mother's age	0.035	(-0.107, 0.177)	0.632	0.032	(-0.110, 0.174)	0.654	0.032	(-0.109, 0.174)	0.655
Mother completed high school	2.776	(0.670, 4.883)	**0.010**	2.831	(0.726, 4.936)	**0.008**	2.922	(0.814, 5.029)	**0.007**
Child's age (months)	0.037	(-0.106, 0.179)	0.613	0.036	(-0.106, 0.179)	0.616	0.040	(-0.102, 0.183)	0.578
Child's sex (girl)	-0.273	(-1.810, 1.265)	0.728	-0.270	(-1.806, 1.266)	0.731	-0.282	(-1.817, 1.254)	0.719
Child has sibling(s)	-1.234	(-3.476, 1.008)	0.281	-1.172	(-3.415, 1.070)	0.306	-1.115	(-3.359, 1.128)	0.330
Mother's participation in non-agricultural labor	-0.178	(-1.867, 1.512)	0.837	-0.223	(-1.913, 1.468)	0.796	-0.193	(-1.881, 1.496)	0.823
Father's employment status	0.865	(-0.808, 2.538)	0.311	0.969	(-0.713, 2.651)	0.259	0.934	(-0.749, 2.618)	0.277
Owns books	1.960	(0.226, 3.694)	**0.027**	1.969	(0.238, 3.701)	**0.026**	1.967	(0.237, 3.698)	**0.026**
Access to improved sanitation facilities	0.864	(-1.618, 3.347)	0.495	0.832	(-1.648, 3.312)	0.511	0.860	(-1.617, 3.337)	0.496
Constant	18.182	(10.394, 25.971)	0.000	17.683	(9.851, 25.515)	0.000	17.820	(9.991, 25.650)	0.000
Group-level variance	2.534	(0.520, 10.854)		2.435	(0.551, 10.760)		2.207	(0.451, 10.792)	
Residual variance	49.095	(41.959, 57.445)		49.000	(41.873, 57.329)		48.978	(41.854, 57.315)	
Intraclass Correlation Coefficient (ICC)	0.049	(0.012, 0.186)		0.047	(0.011, 0.185)		0.043	(0.009, 0.186)	
